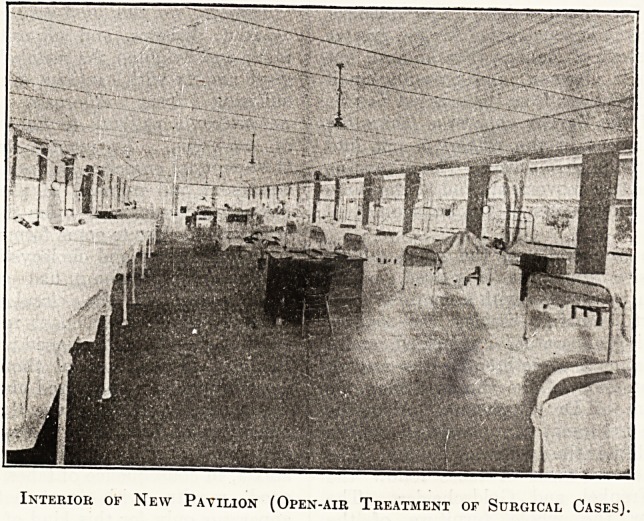# Open-Air Wards in Australia

**Published:** 1913-09-20

**Authors:** 


### OPEN-AIR WARDS IN AUSTRALIA.

### The New Surgical Pavilion at Brisbane Hospital, Queensland.

In view of The Hospital's recent remarks on "the passing of the closed ward" it is interesting to learn from the 1912 Report of the Brisbane

The New (Surgical) Pavilion.

Hospital of the new open-air ward of twenty-six beds which has been opened there. The principle of the open-air ward is not a new one in Australia, judging by the growing popularity of the country hospitals for children and surgical cases; and of the verandah system which provides open-air treatment on such an extensive scale at Queen Mary's Hospital for Sick Children at Carshalton (described in The Hospital of July 26, 1913); and by the increasing popularity of full-size balconies, of which the new ones at the Royal Sussex Hospital, Chichester, are an example, it is a reasonably safe prophecy that the open-air ward will soon become a permanent institution in this country.

As the above brief description suffices to show, the success of an open-air ward depends a good deal upon the nicety of the mechanical contrivances with which its collapsible walls or screens are fitted. Among the many attempts to secure the apparently simple blinds or screens necessary we recently heard an adaptation of the ordinary shop-window shutter spoken of very well by a medical superintendent. But there is still plenty of room for experiment, and there is not a hospital or sana-

Interior of New Pavilion (Open-air Treatment of Surgical Cases).

and has been applied to the smaller institutions for some years past.

The open-air ward in the Brisbane Hospital, of which we give two illustrations from the Report, is made so that the large panels are filled with canvas blinds fitted to American spring rollers, which are nearly always "up." They can, however, be lowered past the floor level (which is raised above that of the ground), and the ward can thus be made snug in windy weather. "This plan is worth a moment's consideration and regard," writes Mr. A. P. Payne, who is secretary to the Brisbane Hospital, and to whom we are indebted for the information and the illustrations, "but (he adds) you cannot in England imagine what a joy it is here." As a matter of fact, torium that has built a shelter which has not something to teach in this respect. We invite descriptions and illustrations of such blinds from our readers.

## Figures and Tables

**Figure f1:**
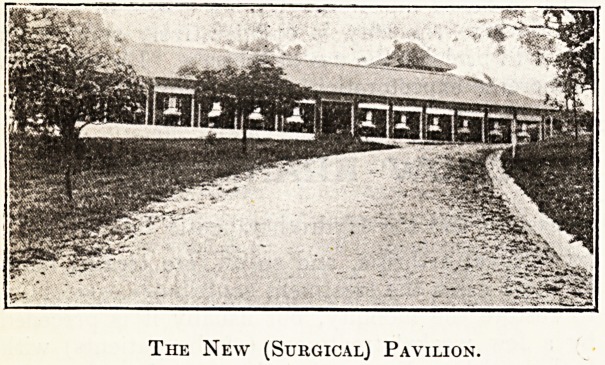


**Figure f2:**